# Clinical Cases

**Published:** 1888-06

**Authors:** Stuart Close

**Affiliations:** Brooklyn, N. Y.


### CLINICAL CASES.

STUART CLOSE, M. D., BROOKLYN, N. Y.

CASE III.—*Diphtheria.*—Mrs. J. E. B., aged twenty-nine. Blonde. For nearly a week has felt a sense of impending illness ; tired, languid, general depression.

*Status præsans :*

1. Last night, sleepless, restless, turning frequently, *because bed felt so hard.*

2. Heat came on yesterday at two P. M., and continued all night ; *chilly on movement*, cheeks flushed and hot.

3. Aching of the flesh, all over body ; " aches all over."

4. Sensation of great *fatigue.*

5. Dry sensation in throat, with thirst for *cold drinks*, which relieve.

6. Mouth full of slightly viscid saliva, tasting salty, running out and wetting pillow during sleep.

7. Frequent swallowing.

8. Sharp pains through tonsils, aggravation right side, and on swallowing.

9. Throat externally sensitive, aggravation right side.

10. The pain and soreness began on the right side, and extended to the left.

11. Right hand and arm " go to sleep " easily.

*Examination :*

12. Tonsils swollen, red, shining.

13. Membrane on tonsils light yellow in color, most on right side, diffused, *in points*, about the size of a large pinhead, " looking like lid of a pepper-box." Twenty-five or thirty of these points on the right tonsil, about half as many on the left. Also points, and larger, irregular patches on posterior wall and pillars of the pharynx.

14. Offensive odor of breath, characteristic of the graver forms of the disease.

Taking into consideration, as the most important points, the peculiar color and disposition of the membrane, the increase of saliva, the frequent swallowing, and the external sensitiveness of the throat, I placed on the tongue of the patient a single dose of Lac. canin.$^{45m}$ The next morning the patient met me at the door of her room with the exclamation, "I'm *ever* so much better!" She had slept nearly all night, waking only a few times, and in the morning felt refreshed and rested. The "tired feeling" was gone. She had no sensation as if the bed were too hard; no fever, nor chilliness on moving. The "aching all over" had disappeared, as had also the unusual accumulation of saliva, the frequent swallowing, and the external sensitiveness of the throat. On examination the tonsils were found much less swollen, no membrane whatever being visible on the left tonsil, and only about half as much as formerly on the right. The shiny, glistening appearance of the inflamed part was nearly gone.

She informed me that early in the morning, after coughing and scraping the throat several times, she expectorated two pieces of membrane, one nearly half an inch in diameter. Both pieces were quite thick, light-yellow in color, one surface being red and bloody, as if torn from a raw surface. After this she had a raw feeling in the throat. She remarked that she felt quite well unless she tried to walk about, and then she felt as if she had been sick a month, so weak and trembling were her limbs. She thought "those little pills were just wonderful."

I reproved her for her imprudence in getting up, and directed absolute rest in bed for at least two days. She declared, however, that she could not; that she had no one to help her; that her husband's meals must be cooked, etc. The result was that when I called next day I found her feeling much worse. She had been restless during the night, and there was very little if any further improvement in the throat. I now insisted upon her going at once to bed, which she did, letting her husband, who "thought, because she got better so quick, there wasn't much the matter," shift for himself. In three days she was entirely well without further medicine.

The patient received only the one dose of the remedy, which was potentized by Dr. Fincke, of Brooklyn.

It may be of interest and value to note, for further confirmation the presence and removal of several symptoms not recorded in the pathogenesis of Lac. can.:

1. Sensation as if bed were too hard. (Arnica, Baptisia.)

2. Chill on movement. (Bry., Nux, Rhus, Sep., Sil., etc.)
3. Aching all over.
4. Sensation of great fatigue.
5. Desire for and relief from *cold* drinks. (Lycop.)

